# Morphofunctional Features of the Immune System Response to Sublethal Hypoxic Load in Hypoxia-Tolerant and Hypoxia-Susceptible Animals

**DOI:** 10.3390/biomedicines13123022

**Published:** 2025-12-10

**Authors:** Maria Kirillova, Dzhuliia Dzhalilova, Mariia Zubareva, Nikolai Fokichev, Olga Makarova

**Affiliations:** 1 Laboratory of Inflammatory Immunomorphology, Avtsyn Research Institute of Human Morphology of Federal State Budgetary Scientific Institution «Petrovsky National Research Centre of Surgery», Moscow 117418, Russia; marusyasilina99@yandex.ru (M.K.); fokichev.n@mail.ru (N.F.); makarov.olga2013@yandex.ru (O.M.); 2 Department of Hystology, Petrovsky Medical University, Moscow 119435, Russia; 3 Faculty of Biology, Lomonosov Moscow State University, Moscow 119234, Russia; mariya.zubareva22@gmail.com

**Keywords:** hypoxia tolerance, immune system, immune cells

## Abstract

**Background/Objectives**: The most commonly used method for determining tolerance to oxygen deficiency is applying a sublethal hypoxic load (SHL) in a decompression chamber at an altitude of 11,500 m. The aim of this study was to identify the morphofunctional characteristics of the immune system’s response to SHL in animals with different tolerances to oxygen deficiency. **Methods**: This study was conducted on male Wistar rats. Resistance to SHL was determined at a critical altitude (11,500 m) once in a decompression chamber. To study the features of the reaction to SHL, morphological and morphometric methods, flow cytometry, and real-time PCR were used. **Results**: One month after SHL, rats susceptible to hypoxia, in comparison with tolerant ones, demonstrated higher numbers of cytotoxic T-lymphocytes and NK cells in the peripheral blood, thymic bodies in the cyst-like cavities formed in the thymus, and wide germinal centers of lymphoid nodules in the spleen. At the same time, rats tolerant to hypoxia demonstrated higher numbers of B-lymphocytes in the peripheral blood and a narrow marginal zone of lymphoid nodules in the spleen. In addition, animals susceptible to hypoxia demonstrated higher expression levels of proinflammatory cytokines *Il1b* and *Tnfa* in peripheral blood leukocytes in comparison with tolerant animals. **Conclusions**: This indicates that the immune systems of tolerant and susceptible animals respond differently to oxygen deprivation during SHL, and that the manifestations of this effect persist for at least a month afterward. The obtained data should be taken into account when conducting experiments with reference to organisms’ initial hypoxia tolerance.

## 1. Introduction

It was demonstrated that tolerance to low oxygen concentrations varies across populations and depends on sex, age, biorhythms, and the presence of comorbidities [1,2,3,4]. At present, the most commonly used method for determining hypoxia tolerance is exposure in a decompression chamber at an altitude of several thousand meters. In humans, this approach is used for the selection of individuals in extreme professions, such as pilots and astronauts, with the “altitude” typically not exceeding 6500 m [5,6,7,8]. Since laboratory animals are generally more tolerant to hypoxia than humans, higher “altitudes” are used to determine their tolerance to low oxygen levels. For example, the critical “altitude” for C57Bl/6 mice is considered to be 10,000 m [9], and for Wistar rats, it is 11,500 m [10]. The primary parameter used by researchers to distinguish organisms tolerant and susceptible to hypoxia is the “gasping time”—the time spent at a critical “altitude” before assuming a lateral position and the appearance of signs of asphyxia. Such exposure to hypoxia in vivo leads to alterative and inflammatory changes in internal organs, complicating the application of this approach to determining the tolerance of healthy individuals and patients to oxygen deprivation [11].

In studies concerning hypoxic tolerance features in animals, their tolerance to oxygen deficiency is determined in a decompression chamber, and the experiments themselves are carried out no earlier than one month after the hypoxic exposure [12]. We previously demonstrated that a sublethal hypoxic load (SHL) when determining tolerance to oxygen deficiency provides an immunomodulatory effect on cytokine production by blood cells, which persists even one month after the exposure [13]. In assessments of the spontaneous and hypoxia- and mitogen complex-stimulated (lipopolysaccharide (LPS), phytohemagglutinin, and concanavalin A) production of IL-1β and IL-10 by peripheral blood cells 2 weeks before and 1 month after exposure to SHL, it was revealed that the response to stimulation by both hypoxia and the complex mitogen after the SHL differed from the initial level, while a proinflammatory phenotype formed in susceptible animals. This was confirmed by studies describing the more severe course of inflammatory processes in mice and rats with low oxygen tolerance [14]. However, specific immune system organ and cell responses in hypoxia-tolerant and hypoxia-susceptible animals to the SHL used to determine tolerance to oxygen deficiency have not been characterized in the literature.

The aim of this study was to identify the morphofunctional characteristics of the immune system’s response to an SHL in animals with different tolerance to oxygen deficiency.

## 2. Materials and Methods

### 2.1. Animals

This study was conducted on 2–3-month-old male Wistar rats (*n* = 30), weighing 250–300 g, obtained from the Stolbovaya branch of the Scientific Center for Biomedical Technologies of the Federal Medical and Biological Agency of Russia. The experimental unit was a single animal. Rats were housed at a regulated room temperature of 25 ± 2 °C under a 12:12 h light–dark cycle and 40–50% relative humidity with ad libitum access to water and food (Char; JSC Range-Agro, Turakovo, Russia). The principles of the European Convention for the Protection of Vertebrate Animals used for Experimental Research (Strasbourg, 1986) and Directive 2010/63/EU of the European Parliament and of the European Union Council were followed. Approval for the study was obtained from the Petrovsky Russian Scientific Center of Surgery Bioethics Committee (Protocol No. 11 dated 20 December 2024). All procedures were performed in accordance with the ‘Animal Research Reporting of In Vivo Experiments’ (ARRIVE) guidelines and the AVMA euthanasia guidelines 2020. According to the literature, hypoxia tolerance depends on an animal’s sex. It was demonstrated that hypoxia-tolerant organisms are predominantly found among females, whereas susceptible and normal organisms are predominantly among males, with sex differences also observed in the morphofunctional state of the immune system [15,16]. To eliminate sex-based differences, this study was performed exclusively on male rats. The rats were randomly divided into experimental groups, each containing a minimum of five animals.

### 2.2. Determination of Hypoxia Tolerance and SHL Modeling

**Figure 1:**
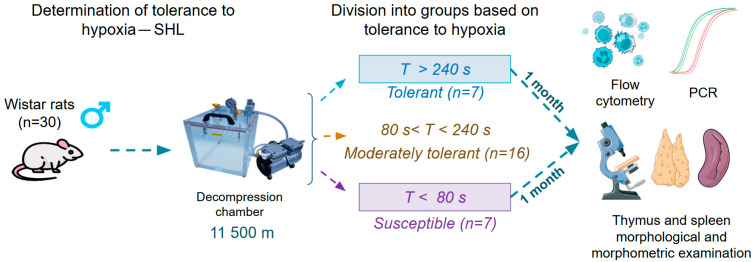
The experimental plan.

The rats’ resistance to the SHL was determined at a critical altitude (11,500 m) once in a decompression chamber based on the “gasping time” before assuming a lateral position and the appearance of asphyxia signs [4,10] (Figure 1). Two groups of rats were identified: hypoxia-tolerant (*n* = 7) with a “gasping time” of more than 240 s and hypoxia-susceptible (*n* = 7) with a “gasping time” of less than 80 s. Animals were sacrificed one month after the SHL in a decompression chamber by intramuscular administration of zoletil at a dose of 15 mg/kg (Virbac Sante Animale, Carros, France). According to the literature, a one-month time period is necessary to mitigate the negative effects of acute hypoxia (at a critical altitude) on the organism [12]. Animals moderately tolerant to oxygen deficiency (*n* = 16) with a “gasping time” of 80 to 240 s were excluded from the experiment after their hypoxia resistance was determined. For each animal, different researchers were involved as follows: The first researcher (DD) modeled the SHL and was responsible for sacrificing the animals. This researcher was the only person aware of the group allocation. The second and third researchers (MK, MZ) performed the real-time PCR, flow cytometry, and morphological and morphometric studies.

### 2.3. Flow Cytometry

Venous blood was collected from the jugular veins of the animals into test tubes containing EDTA as an anticoagulant (concentration of 5 mg/mL). The absolute and relative lymphocyte counts in the blood were determined using a Mindray BC-2800Vet automatic hematology analyzer (Mindray, Shenzhen, China). The number of main lymphocyte subpopulations in the peripheral blood was analyzed using a Cytomics FC 500 flow cytometer (Beckman Coulter, Brea, CA, USA) with the following fluorescently conjugated antibodies (Thermo Fisher Scientific, Waltham, MA, USA): FITC- (Fluorescein Isothiocyanate), PE- (Phycoerythrin), and PE-Cy7-conjugated (Phycoerythrin-Cyanine 7) anti-Rat CD3 (T-lymphocyte marker), anti-Rat CD4 (T-helper cell marker), anti-Rat CD8a (cytotoxic T-lymphocyte marker), anti-Rat CD45R (B-lymphocyte marker), and anti-Rat CD314 (NK cell marker). The relative contents of T-lymphocytes (CD3+ cells), B-lymphocytes (CD45R+ cells), and NK cells (CD314+) were calculated relative to the total lymphocyte count. The proportions of T-helpers (CD3+CD4+ cells) and cytotoxic T-lymphocytes (CD3+CD8a+ cells) were calculated in relation to the absolute number of T-lymphocytes. Lymphocytes were gated based on their morphology using forward- versus side-scatter (FSC-SSC) dot plots. This approach was chosen because the samples were not fixed, erythrocytes were lysed, and flow cytometric analysis was performed within 4–6 h after sample collection, with cells kept at room temperature throughout. The gating strategy is presented in Supplementary File S1. For the flow cytometry data analysis, FlowJo software (v10.10) was used (Becton, Dickinson & Company, Franklin Lakes, NJ, USA).

### 2.4. Real-Time PCR in Peripheral Blood Leukocytes

Venous blood was collected from the jugular veins, erythrocytes were lysed, and IntactRNA (Eurogen, Moscow, Russia) was added to fix the RNA, which was stored at −80 °C. Total RNA was subsequently isolated from leukocytes using the RNA Solo kit (Eurogen, Moscow, Russia), and reverse transcription was performed using the MMLV RT Kit (Eurogen, Moscow, Russia). The mRNA expression levels of *Nfkb*, *Il1b*, *Tnfa*, *Tgfb*, and *Il10* in leukocytes were determined with the qPCRmix-HS SYBR (Eurogen, Moscow, Russia) and primers synthesized by Evrogen (Supplementary Table S1) by the RT-PCR method relative to the *Gapdh* expression level on a DTprime amplifier (DNA-Technology, Moscow, Russia). The relative mRNA levels for each gene were calculated by the ΔΔCq method [17].

### 2.5. Thymus and Spleen Morphological and Morphometric Examination

For the morphological examination, the thymus and spleen were fixed in Bouin’s fluid for 24 h and embedded in paraffin; then histological sections were prepared and stained with hematoxylin and eosin. The volumetric density ratio of the thymic cortex to the medulla (magnification 200) and the number of thymic bodies (magnification 400) were assessed using a light microscope using the point counting method. Using the same method, the volumetric densities of the white and red pulp, lymphoid nodules, and periarteriolar lymphoid sheath (PALS; magnification 200), as well as the germinal centers and marginal zones of the lymphoid nodules of the spleen (magnification 400), were determined.

### 2.6. Statistics

Statistical analysis was performed using Statistica 8.0 (StatSoft, Inc., Tulsa, OK, USA). The distribution of the parameters was determined using the Kolmogorov–Smirnov test. Because the data were not normally distributed, the significance of differences between parameters was determined using the nonparametric Mann–Whitney test. Data are expressed as the median (Me) and interquartile range (25–75%). Differences were considered statistically significant at *p* < 0.05.

## 3. Results

### 3.1. Flow Cytometry

**Figure 2:**
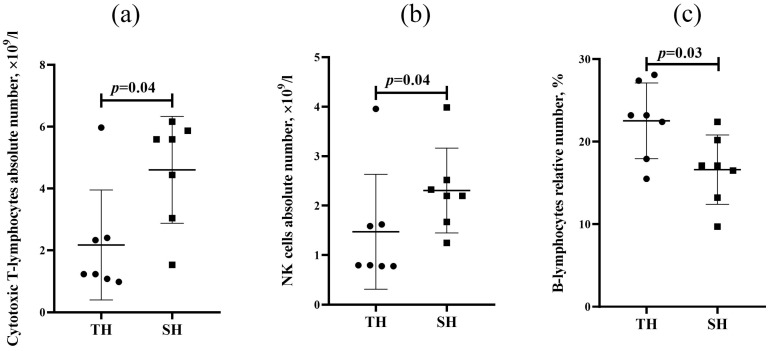
Absolute (**a**,**b**) and relative (**c**) numbers of cytotoxic T-lymphocytes (**a**), NK cells (**b**), and B-lymphocytes (**c**) in the peripheral blood of hypoxia-tolerant (TH) and hypoxia-susceptible (SH) rats after the SHL. Circles and squares represent the parameter values for each animal in the group. Me (25–75%). *p*—statistically significant differences, Mann–Whitney test.

The absolute numbers of cytotoxic lymphocytes (Figure 2a) and NK cells (Figure 2b) were higher in hypoxia-susceptible rats, while the relative number of B-lymphocytes was higher in hypoxia-tolerant animals (Figure 2c). No statistically significant differences in the absolute and relative numbers of T-lymphocytes and T-helpers were revealed between hypoxia-tolerant and hypoxia-susceptible animals (Figure S1).

### 3.2. Cytokine Expression Levels in Peripheral Blood Leukocytes

**Figure 3:**
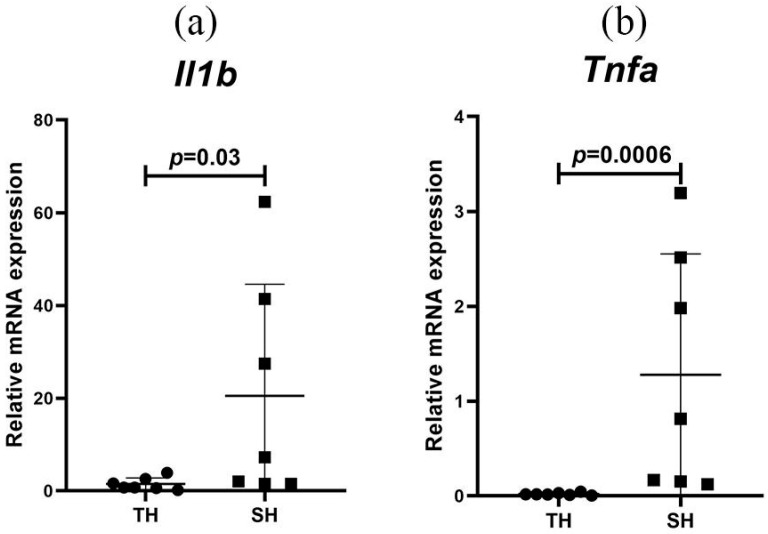
*Il1b* (**a**) and *Tnfa* (**b**) expression levels in peripheral blood leukocytes of hypoxia-tolerant (TH) and hypoxia-susceptible (SH) rats after the SHL. Circles and squares represent the parameter values for each animal in the group. Me (25–75%). *p*—statistically significant differences, Mann–Whitney test.

One month after the SHL, higher expression levels of proinflammatory cytokine genes *Il1b* and *Tnfa* were detected only in hypoxia-susceptible animals (Figure 3). At the same time, *Nfkb*, *Tgfb*, and *Il10* expression levels in peripheral blood leukocytes did not differ significantly (Figure S2).

### 3.3. Morphological and Morphometric Examination of the Thymus

**Figure 4:**
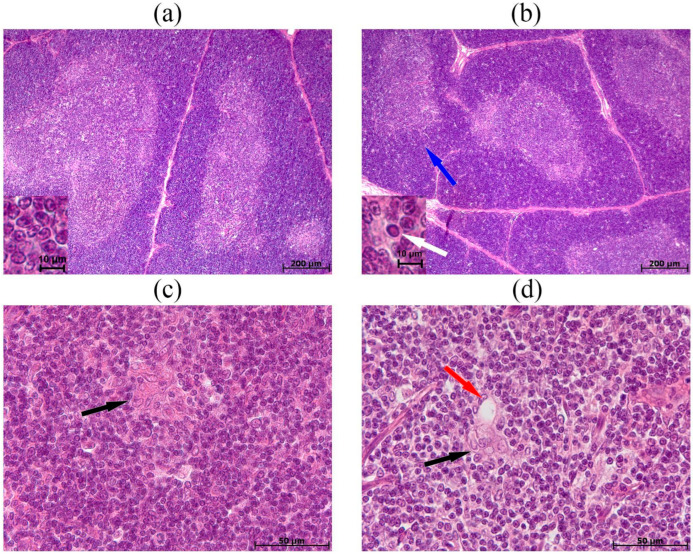
Thymus morphological characteristics in hypoxia-tolerant (**a**,**c**) and hypoxia-susceptible (**b**,**d**) rats after the SHL: (**a**)—the boundaries between the cortex and medulla are clear, and the thymic cortex is densely populated with lymphocytes (inset); (**b**)—the boundaries between the cortex and medulla are focally unclear (blue arrow), and in the cortex there is a small number of diffusely scattered pale zones in which dying thymocytes are located (inset, white arrow); (**c**)—thymic bodies in the form of accumulated epithelial cells (black arrow); (**d**)—thymic bodies in the form of accumulated epithelial cells (black arrow) and cyst-like cavities (red arrow) developing. Hematoxylin and eosin staining.

Morphological examination of the thymus in hypoxia-tolerant animals revealed cortices densely populated with lymphocytes. The subcapsular zone contained 5–7 rows of lymphoblasts. The boundaries between the cortex and medulla were distinct (Figure 4a). The medulla consisted of lymphocytes and epithelial cells in an approximate ratio of 1:1. Thymic bodies composed of 3–5 epithelial cells (Figure 4c), as well as single ones with keratinization, were also revealed. In one animal from this group (14.3%), thymic bodies in the form of developing cyst-like cavities were detected. In contrast, in hypoxia-susceptible rats, the cortex predominated over the medulla, and their boundaries were focally indistinct (Figure 4b). Additionally, a small number of diffusely scattered pale zones were observed in the cortex, containing dying thymocytes (Figure 4b, inset). Thymic bodies were detected in the form of cyst-like cavities in five animals from this group (71.4%) (Figure 4d). As in rats tolerant to hypoxia, in susceptible ones, thymic bodies consisting of 3–5 cells and with keratohyalin deposits were detected.

**Figure 5:**
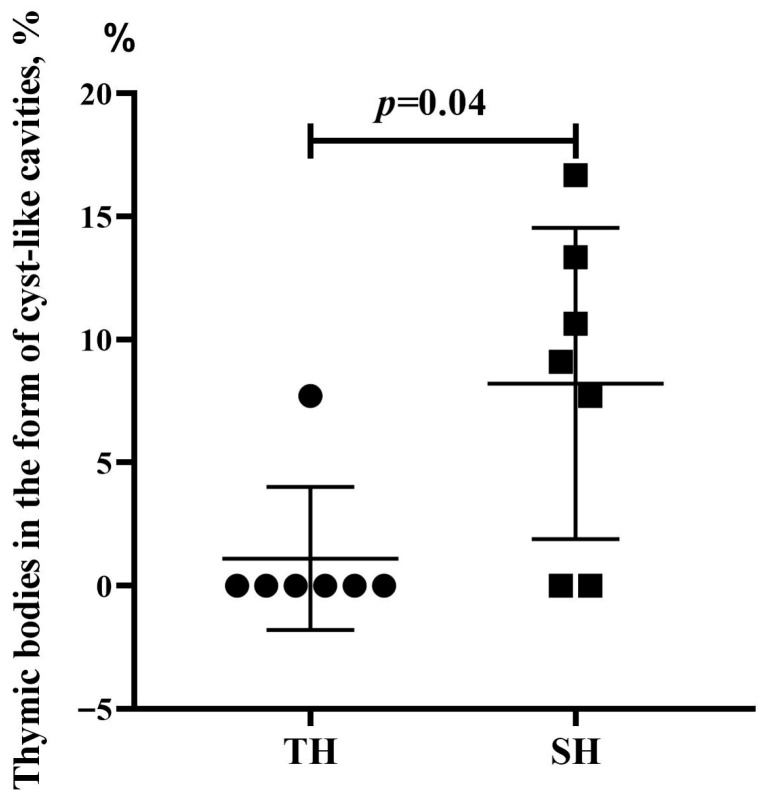
The numbers of thymic bodies in the form of cyst-like cavities in hypoxia-tolerant (TH) and hypoxia-susceptible (SH) rats after the SHL. Circles and squares represent the parameter values for each animal in the group. Me (25–75%). *p*—difference’s statistical significance, Mann–Whitney test.

Thymus morphometric examination revealed no statistically significant differences in the volumetric density of the cortex and medulla between the groups (Figure S3). Furthermore, the number of thymic bodies—whether present as clusters of 3–5 cells, comprising more than 5 cells, or containing keratohyalin deposits—did not differ significantly between hypoxia-tolerant and hypoxia-susceptible rats (Figure S3). However, the number of thymic bodies in the form of cyst-like cavities was statistically significantly higher in susceptible rats in comparison to the tolerant ones (Figure 5).

### 3.4. Spleen Morphological and Morphometric Examination

**Figure 6:**
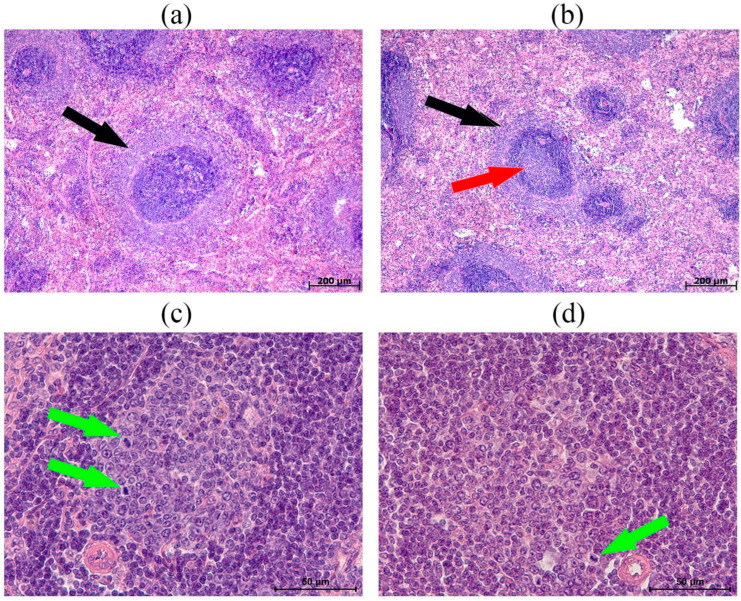
Spleen morphological characteristics in hypoxia-tolerant (**a**,**c**) and hypoxia-susceptible (**b**,**d**) rats after the SHL: (**a**)—a lymphoid nodule without a germinal center and with a wide marginal zone (black arrow); (**b**)—a lymphoid nodule with a germinal center (red arrow) and a narrow marginal zone (black arrow); (**c**,**d**)—germinal centers with densely packed lymphoblasts and lymphocytes, as well as mitoses (green arrows). Hematoxylin and eosin staining.

Spleen morphological examination in hypoxia-tolerant rats revealed a predominance of red pulp. The white pulp consisted of lymphoid nodules with small germinal centers containing densely packed lymphoblasts and lymphocytes. Single mitoses were observed (Figure 6c). Lymphoid nodules without germinal centers were also identified (Figure 6a). The marginal zone of the lymphoid nodules was wide (Figure 6a), and the T-dependent PALS zone was clearly visible.

In hypoxia-susceptible rats, the splenic morphology also showed a predominance of red pulp. The white pulp consisted of lymphoid nodules with germinal centers (Figure 6b,d) and a narrow marginal zone. The PALS zone was clearly visible.

**Figure 7:**
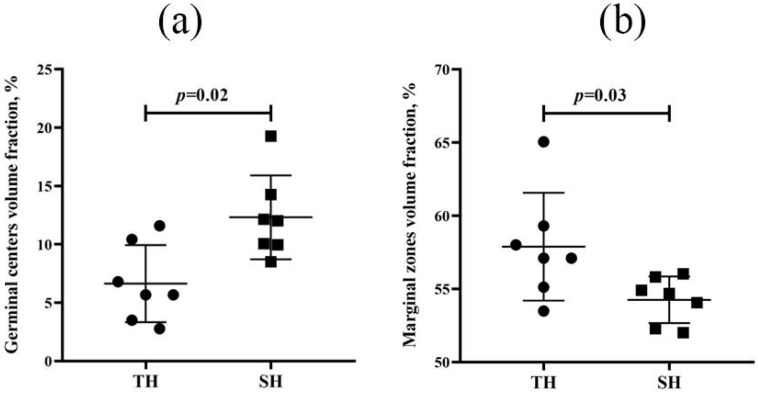
Volume density of germinal centers (**a**) and marginal zones (**b**) in hypoxia-tolerant (TH) and hypoxia-susceptible (SH) rats’ spleen lymphoid nodules after the SHL. Circles and squares represent the parameter values for each animal in the group. Me (25–75%). *p*—statistical significance of differences, Mann–Whitney test.

Spleen morphometric examination revealed no statistically significant differences in the volumetric density of the red and white pulp (Figure S4) or in lymphoid nodules or the PALS zone (Figure S4) between tolerant and susceptible rats. However, hypoxia-susceptible animals had wider germinal centers of the lymphoid nodules (Figure 7a), while hypoxia-tolerant animals had wider marginal zones (Figure 7b).

## 4. Discussion

The thymus is the central organ of the immune system, consisting of connective tissue stroma, epithelial reticulum, and a predominant population of lymphocytes [18]. The thymus’s primary function is to ensure the maturation, selection, and differentiation of T-lymphocytes. These processes are mediated by thymic stromal lymphopoietin, synthesized by epithelial cells [19,20]. During selection, the expression of anti-apoptotic factors in cortical thymocytes decreases, making them more susceptible to apoptosis [21]. In this study, one month after the SHL, differences in thymus morphology were detected: in hypoxia-tolerant rats, the thymus structure was normal, while in hypoxia-susceptible rats, dying thymocytes and focally unclear boundaries between the cortex and medulla were detected, indicating active T-lymphocyte migration. Additionally, thymic bodies with involutional changes, including cyst-like cavity development, were detected in animals of this group. The thymus’s stereotypical response to various stressors (emotional, pain, informational, mobilization, or hypoxic stress) is morphologically characterized by accidental involution with a progressive decrease in the mass, volume, and functional activity of the thymus. Accidental involution develops as a manifestation of adaptation syndrome [22]. This response is mediated by the cytokines IL-12, IL-18, IFNγ, and IL-33 [23,24] and cortisol [25], leading to lymphocyte death. LPS also triggers acute thymus atrophy. It was demonstrated that during a systemic inflammatory response induced by LPS administration, a disruption of the clear boundaries between the lobules cortex and medulla was observed in the thymus [26,27], including in animals with different tolerance to oxygen deficiency [14]. Thymus involution leads to a decrease in the production of naive T-cells and a limitation of the T-cell receptor repertoire, potentially weakening the immune system [28]. Thus, in hypoxia-susceptible animals after SHL, the production of proinflammatory cytokines increases, since we previously identified a proinflammatory phenotype in them [13], which leads to accidental thymus involution.

Furthermore, higher absolute numbers of cytotoxic T-lymphocytes (CD3+CD8+) and NK cells (CD314+) were revealed in the peripheral blood of hypoxia-susceptible rats, which could be associated with trained immune responses to the damaging effects of SHL characteristics, resulting in DAMP (Danger-Associated Molecular Pattern) development. Recent studies demonstrated that NK cells can acquire a trained phenotype after pattern-recognition receptor stimulation, providing protection upon re-exposure [29]. Upon the initial stimulation, these cells undergo modifications that enable them to mount a more effective response upon repeated exposure [30]. Hypoxia plays a significant role in the development of the trained immune cells phenotype; changes in the cells’ functional state are associated with their metabolism restructuring, with a shift from oxidative phosphorylation to aerobic glycolysis, as well as with HIF-1α (Hypoxia-Inducible Factor) and mTOR (mammalian Target of Rapamycin) signaling pathway activation [31]. Trained immunity relies on epigenetic reprogramming, which influences the functional state of cells without causing mutations [32]. This leads to the development of an adaptive state that increases the organism’s resilience to repeated stimuli. However, in case of incorrect activation, trained immune programs can become maladaptive, as in post-septic immune paralysis or autoinflammatory diseases.

The lymphoid nodules’ marginal zone, located at the spleen red and white pulp junction, consists mainly of B-lymphocytes and dendritic cells, which are responsible for antigen capture, migration, and presentation to T-lymphocytes [33]. The narrower marginal zone of the spleen lymphoid nodules in hypoxia-susceptible rats reflects active processes of lymphocyte migration and death. At the same time, the spleen lymphoid nodule germinal center expansion in animals of this group indicates high B-lymphocyte proliferative activity in response to antigenic exposure. Under physiological conditions, the germinal centers of splenic lymphoid nodules present a hypoxic microenvironment due to high oxygen consumption during dendritic cell–B-lymphocyte interactions [34]. Thus, the more pronounced changes in the splenic B-zone of hypoxia-susceptible animals after the SHL are probably associated with the predominant activation of the humoral immunity component, consistent with prior data from an LPS-induced systemic inflammation model in animals with different tolerance to oxygen deficiency [14].

Morphofunctional immune differences between hypoxia-tolerant and hypoxia-susceptible animals may originate from their distinct energy metabolism. Specifically, tolerant rats display enhanced mitochondrial biogenesis and function, including increased numbers of mitochondria with ultrastructural specializations (denser cristae, darker matrix), a higher count of small active mitochondria, and greater mitochondrial enzyme activity [35,36]. As major ROS producers, mitochondria influence tolerance both structurally and by modulating oxidative stress levels [37,38]. This metabolic flexibility was further evidenced by experiments with L-arginine and its inhibitor: highly tolerant animals showed a more rapid reconfiguration of oxygen-dependent metabolic pathways for adaptation to nitrate exposure [39].

Genetic factors, particularly polymorphisms in genes involved in the cellular hypoxic response, contribute to individual variation in hypoxia resistance. Research by Zhang et al. (2020) identified associations between several SNPs (*EPAS1* rs675666667, *VEGFA* rs3025039, *PPARA* rs7292407, *EGLN1* rs2153364) and the manifestation of AMS symptoms across different organ systems [40]. While specific genotypes (e.g., GG at rs675666667 and CC at rs3025039) correlate with higher or lower risks of mild AMS subtypes, the practical application of these SNPs as biomarkers is limited due to substantial heterogeneity in allele frequencies across diverse human populations, complicating universal predictive models [41].

According to the literature, studies on hypoxia-tolerant and -susceptible animals typically determine oxygen tolerance using a decompression chamber, with experiments conducted no earlier than one month post-exposure to mitigate the acute negative effects of hypoxia [12]. However, it was previously demonstrated that SHL in a decompression chamber has an immunomodulatory effect on cytokine production by blood cells that persists even a month after exposure [13]. Combined with the morphofunctional features of the immune response identified in animals with different tolerance to hypoxia, the problem of searching for non-invasive markers of tolerance to oxygen deficiency without the use of an SHL and a decompression chamber remains relevant.

HIF-family transcription factors, represented by three α-subunit isoforms—HIF-1α, HIF-2α, and HIF-3α—and the HIF-1β protein, are key regulators of the cellular response to hypoxia. Hypoxia and HIF activation are linked to an increase in the activity of nuclear factor kappa B (NF-κB), which regulates inflammatory processes [42,43,44]. This transcription factor activation occurs as a result of a number of molecules’ sequential phosphorylation [45]. NF-κB then translocates from the cytoplasm to the nucleus and activates the expression of dependent genes encoding proinflammatory cytokines (IL-1β, IL-2, IL-6, TNF-α, etc.), chemokines, acute-phase proteins, and a number of other molecules [46]. Increasing evidence has emerged regarding the relationship between NF-κB and HIF transcription factors [47]. NF-κB is known to influence the HIF-1α protein level and expression directly, under both normoxia and hypoxia, which is explained by the presence of the NF-κB binding site in the proximal promoter region of the *HIF1A* gene [42,43,44]. In our experiment, despite *Nfkb* expression levels not differing one month after the SHL, hypoxia-susceptible animals demonstrated higher expression levels of the proinflammatory cytokines *Il1b* and *Tnfa* in peripheral blood leukocytes compared to tolerant rats, indicating a more pronounced response to oxygen deficiency and proinflammatory phenotype development. Thus, variations in the content and activity of HIF and NF-κB or the expression levels of the genes encoding them, as well as the dependent molecules, may be potential markers of initial hypoxia tolerance in both laboratory animals and future clinical applications.

Individual tolerance to hypoxia is assessed using functional stress tests, including evaluation under normobaric hypoxic conditions with measurements of the hypoxic ventilatory response, oxygen saturation (SpO_2_) dynamics, and exercise tolerance, which is the most informative approach [48]. Screening methods involve voluntary breath-hold (apnea) tests, reflecting integral sensitivity to hypoxia and hypercapnia, as well as heart rate variability analysis as a marker of autonomic regulation [49]. However, the biggest drawback of the characterized markers is that they require hypoxic load. Our recent literature review [50] summarizes markers identified in individuals before high-altitude ascent that indicate a high risk for developing AMS and may also serve as potential markers of hypoxia resistance. Research has identified two distinct types of predictors for AMS. The first type comprises functional indicators like VO_2_max and SpO_2_, measured by relatively straightforward instrumental methods such as ECG and pulse oximetry. The second type involves a complex set of molecular markers (microRNAs, proteins, metabolites), whose identification requires advanced technologies like mass spectrometry and next-generation sequencing. While molecular methods hold significant promise for predictive diagnostics, their current application is limited by two major factors. Firstly, the biological role of many identified molecules in the hypoxic response remains unknown. Secondly, existing clinical studies vary considerably in their design parameters (e.g., the altitude, time, and methods for ascent), making it difficult to compare and consolidate findings. Standardization of experimental conditions is essential for developing reliable predictive models.

The performed study has several limitations. To assess immune system changes in hypoxia-tolerant and hypoxia-susceptible animals, it is important not only to study the morphofunctional alterations in immune organs but also to evaluate lymphocytes’ and macrophages’ functional activity—their produced cytokines, metabolic profile, various subpopulations, and phagocytosis activity. The differences we identified between animals tolerant and susceptible to hypoxia and their responses to SHL may be related to differences in cellular functional activity. Additionally, future studies should assess mitochondrial dysfunction and oxidative stress levels. Despite these limitations, we identified differences confirming the activation of a proinflammatory response in hypoxia-susceptible animals. Our results may serve as a basis for developing a method for determining hypoxia tolerance in laboratory animals and, in the future, in humans. The instrumental and laboratory diagnostic methods used for hypoxia tolerance analysis require expensive equipment and highly qualified personnel. Further research is needed to develop a simple and accessible method for determining hypoxia tolerance and assessing the risk of developing inflammatory diseases in both healthy individuals and patients.

## 5. Conclusions

In this investigation we revealed that the morphofunctional state of the immune system differed one month after an SHL in animals with different hypoxia tolerance. Hypoxia-susceptible rats exhibited higher numbers of cytotoxic T-lymphocytes and NK cells in their peripheral blood, thymic bodies with developing cyst-like cavities, and wide germinal centers of the splenic white pulp lymphoid nodules. Meanwhile, hypoxia-tolerant rats one month after the SHL were characterized by a higher B-lymphocyte number and a narrow marginal zone in comparison to susceptible ones. Additionally, hypoxia-susceptible animals demonstrated higher expression levels of the proinflammatory cytokines *Il1b* and *Tnfa* in peripheral blood leukocytes in comparison to tolerant rats. This indicates that the immune responses of hypoxia-tolerant and hypoxia-susceptible animals differ, and the effects of SHL persist for at least one month. These data highlight the importance of accounting for an organism’s inherent hypoxia tolerance when designing and interpreting experiments.

## Data Availability

The original contributions presented in this study are included in the article/Supplementary Material. Further inquiries can be directed to the corresponding author.

## Supplementary Materials

The following supporting information can be downloaded at https://www.mdpi.com/article/10.3390/biomedicines13123022/s1. File S1. Gating strategy for flow cytometry; Table S1. Oligonucleotide sequences for PCR; Figure S1. Flow cytometry results; Figure S2. *Nfkb*, *Tgfb*, and *Il10* expression levels; Figure S3. Thymus morphometric examination; Figure S4. Spleen morphometric examination.

## Abbreviations

The following abbreviations are used in this manuscript:

SHL	Sublethal hypoxic load
PALS	Periarteriolar lymphoid sheath
LPS	Lipopolysaccharide
HIF	Hypoxia-Inducible Factor
SNP	Single-Nucleotide Polymorphism
AMS	Acute Mountain Sickness
