# Supplementary material for: Morphofunctional Features of the Immune System Response to Sublethal Hypoxic Load in Hypoxia-Tolerant and Hypoxia-Susceptible Animals
Source: Biomedicines. 2025 Dec 10;13(12):3022. doi: 10.3390/biomedicines13123022 (PMC12730225; doi:10.3390/biomedicines13123022)
Supplement: Supplementary file 1 [file biomedicines-13-03022-s001.zip › Supplementary Figures.pdf]

Figure S1. Flow cytometry results

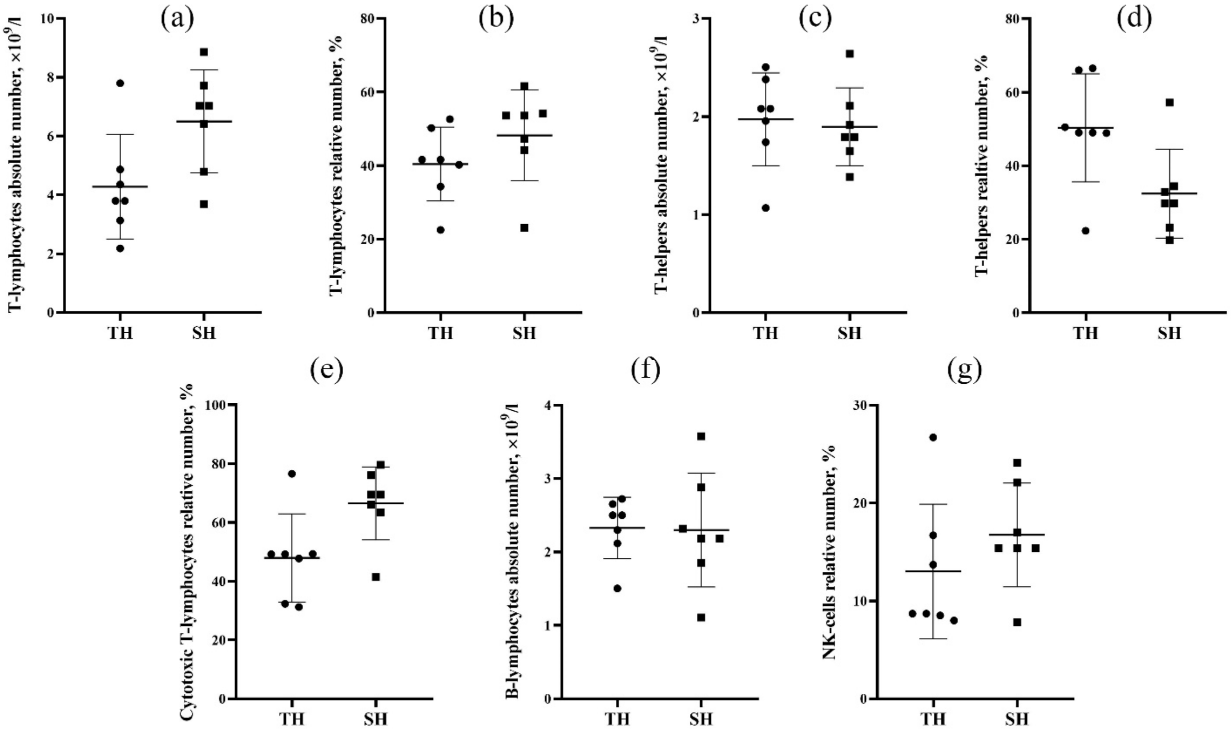

Absolute (a, c, f) and relative (b, d, e, g) numbers of T-lymphocytes (a, b), T helper cells (c, d), cytotoxic T-lymphocytes (e), B lymphocytes (f), and NK cells (g) in the peripheral blood of tolerant (TH) and susceptible to hypoxia (SH) rats after the SHL. Me (25-75%). p – statistically significant differences, Mann-Whitney test

Figure S2. *Nfkb*, *Tgfb* and *Il10* expression levels

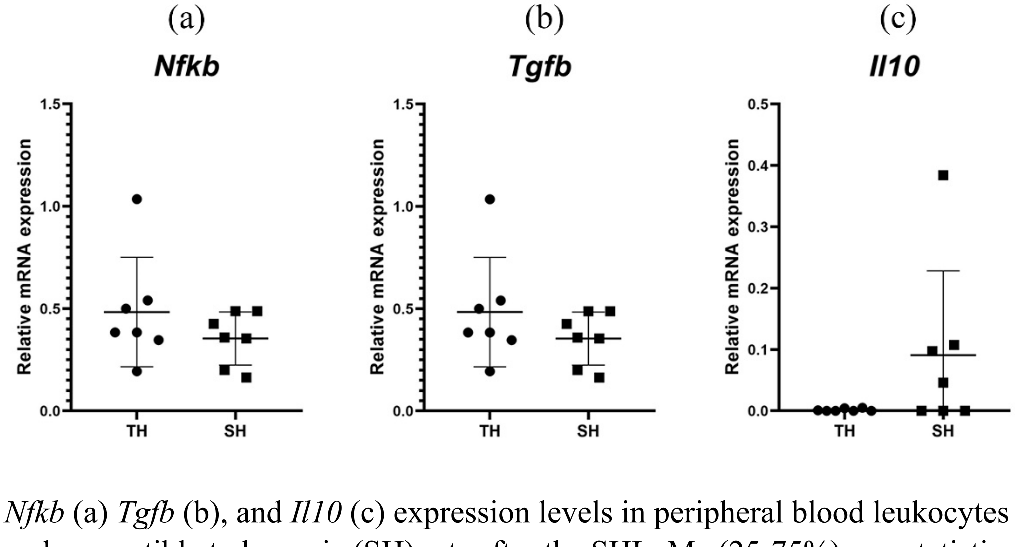

Nfkb (a) *Tgfb* (b), and *Il10* (c) expression levels in peripheral blood leukocytes of tolerant (TH) and susceptible to hypoxia (SH) rats after the SHL. Me (25-75%). p – statistically significant differences, Mann-Whitney test

Figure S3. Thymus morphometric examination

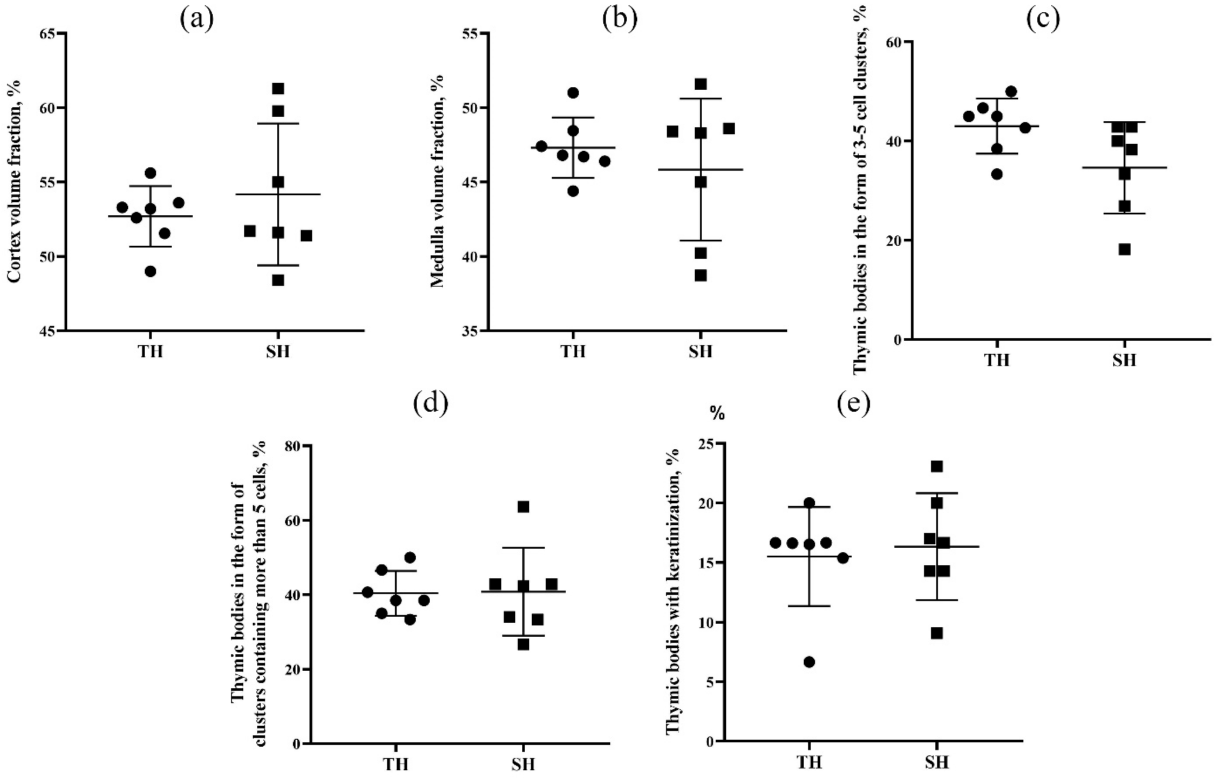

Thymic cortex (a) and medulla (b) volumetric fractions and the thymic bodies number (c, d, e) in tolerant (TH) and susceptible to hypoxia (SH) rats after the SHL. Me (25-75%). p – differences statistical significance, Mann-Whitney test

Figure S4. Spleen morphometric examination

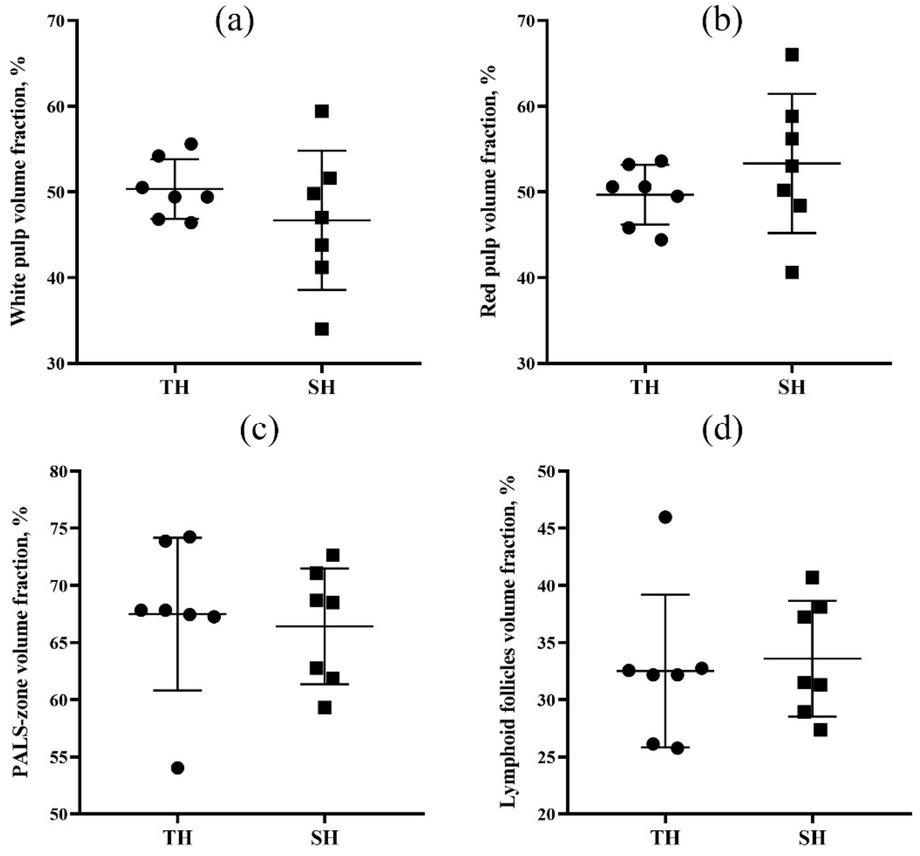

White (a) and red (b) splenic pulp volume fractions PALS zone (c) and lymphoid nodules (d) volume density in tolerant (TH) and susceptible hypoxia (SH) rats after the SHL. Me (25-75%). p – statistical significance of differences, Mann-Whitney test
