# Supplementary material for: Morphofunctional Features of the Immune System Response to Sublethal Hypoxic Load in Hypoxia-Tolerant and Hypoxia-Susceptible Animals
Source: Biomedicines. 2025 Dec 10;13(12):3022. doi: 10.3390/biomedicines13123022 (PMC12730225; doi:10.3390/biomedicines13123022)
Supplement: Supplementary file 1 [file biomedicines-13-03022-s001.zip › Supplementary File S1.pdf]

Gating strategy for flow cytometry

Lymphocytes were selected based on their morphology using forward-versus side-scatter (FSC-SSC) dotplots because the samples were not fixed, erythrocytes were lysed, no more than 4-6 h passed after obtaining the materials and before the study on the flow cytometer, the cells were constantly at room temperature (Figure a,b). Combining FSC-H and anti-CD3 (conjugated with PE) we selected T-lymphocytes (Figure, c,d). Combining anti-CD4 (conjugated with FITC) and anti-CD8a (conjugated with PE-Cy7) we identified T-helpers and cytotoxic T-lymphocytes (Figure e,f).

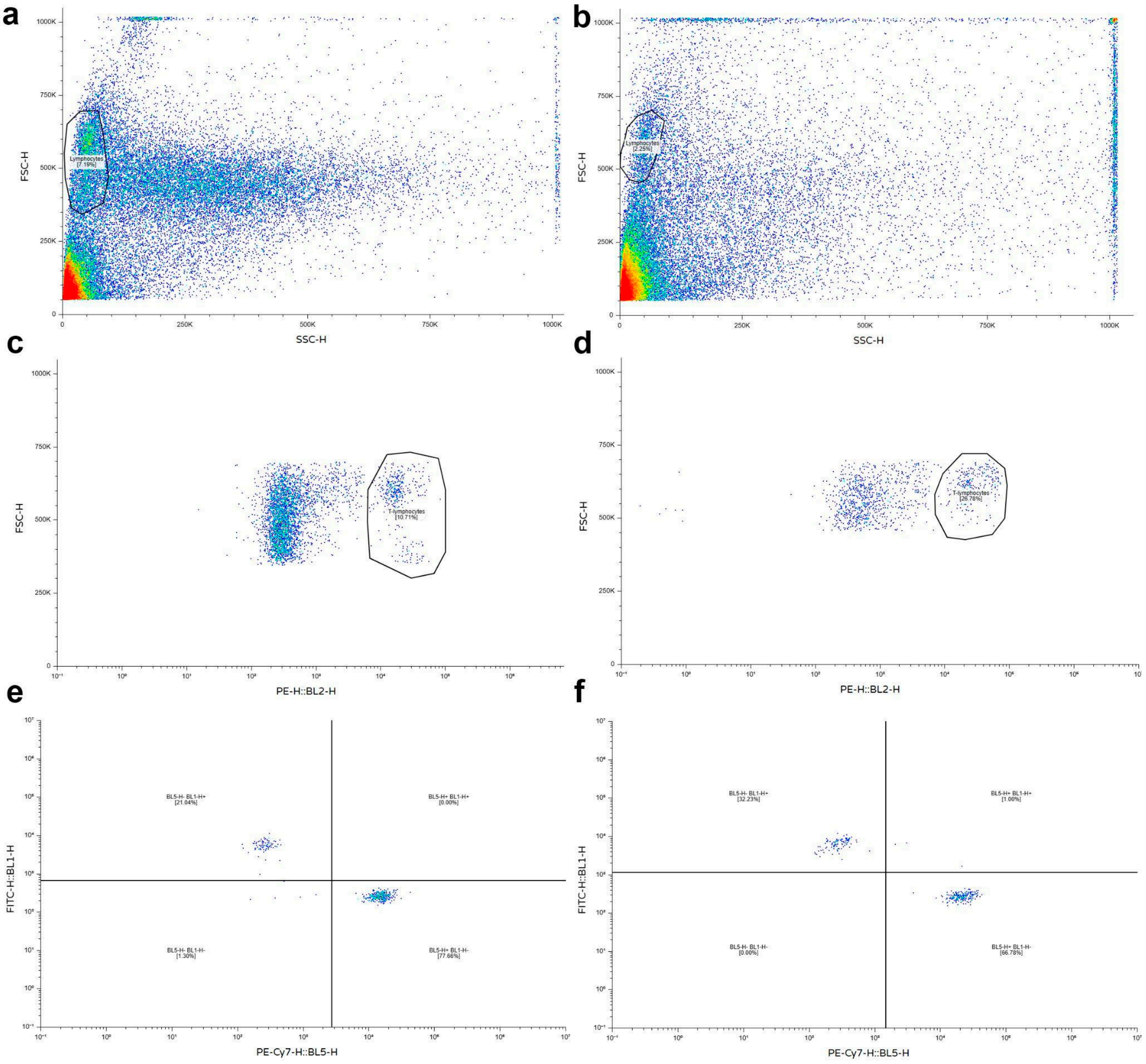

Figure. Gating strategy for flow cytometry of peripheral blood lymphocytes of tolerant (a,c,e) and susceptible to hypoxia (b,d,f) rats after the sublethal hypoxic load. a, b –

gates Lymphocytes; c, d – gates T-lymphocytes; e, f – gates T-helpers (upper left) and cytotoxic T-lymphocytes (lower right).
