# Supplementary material for: Morphofunctional Features of the Immune System Response to Sublethal Hypoxic Load in Hypoxia-Tolerant and Hypoxia-Susceptible Animals
Source: Biomedicines. 2025 Dec 10;13(12):3022. doi: 10.3390/biomedicines13123022 (PMC12730225; doi:10.3390/biomedicines13123022)
Supplement: Supplementary file 1 [file biomedicines-13-03022-s001.zip › Supplementary Table S1.pdf]

Supplementary Table S1. Oligonucleotide sequences for PCR

Gene		Sequences 5'→3'
Gapdh	forward	GCCAGCCTCGTCTCATAGAC
	reverse	CTTGCCGTGGGTAGAGTCAT
Nfkb	forward	GACGATCCTTTCGGAACTG
	reverse	GCATATGCCGTCCTCACAG
Il1b	forward	TTCGACAGTGAGGAGAATGAC
	reverse	CGTCATCATCCCACGAGTCA
Tnfa	forward	GTTCCGTCCCTCTCATACACTG
	reverse	GAAGTTCAGTAGACAGAAGAGCGT
Tgfb	forward	CGTGGCTTCTAGTGCTGACG
	reverse	TGGCGAGCCTTAGTTTGGAC
Il10	forward	CTATGTTGCCTGCTCTTACTG
	reverse	GGCATCACTTCTACCAGGT
